# Development and Evaluation of Reverse Transcription-Loop-Mediated Isothermal Amplification (RT-LAMP) Assay Coupled with a Portable Device for Rapid Diagnosis of Ebola Virus Disease in Guinea

**DOI:** 10.1371/journal.pntd.0004472

**Published:** 2016-02-22

**Authors:** Yohei Kurosaki, N’Faly Magassouba, Olamide K. Oloniniyi, Mahamoud S. Cherif, Saori Sakabe, Ayato Takada, Kenji Hirayama, Jiro Yasuda

**Affiliations:** 1 Institute of Tropical Medicine (NEKKEN), Nagasaki University, Nagasaki, Japan; 2 Laboratoire des Fièvres Hémorragiques en Guinée, Conakry, Guinea; 3 Hôpital National Donka, Service des Maladies Infectieuses et Tropicales, Conakry, Guinea; 4 Université Gamal Abdel Nasser, Conakry, Guinea; 5 Graduate School of Biomedical Sciences and Program for Nurturing Global Leaders in Tropical and Emerging Communicable Diseases, Nagasaki University, Nagasaki, Japan; 6 Research Center for Zoonosis Control, Hokkaido University, Sapporo, Japan; University of Texas Medical Branch, UNITED STATES

## Abstract

Given the current absence of specific drugs or vaccines for Ebola virus disease (EVD), rapid, sensitive, and reliable diagnostic methods are required to stem the transmission chain of the disease. We have developed a rapid detection assay for *Zaire ebolavirus* based on reverse transcription-loop-mediated isothermal amplification (RT-LAMP) and coupled with a novel portable isothermal amplification and detection platform. The RT-LAMP assay is based on primer sets that target the untranscribed trailer region or nucleoprotein coding region of the viral RNA. The test could specifically detect viral RNAs of Central and West African Ebola virus strains within 15 minutes with no cross-reactivity to other hemorrhagic fever viruses and arboviruses, which cause febrile disease. The assay was evaluated using a total of 100 clinical specimens (serum, *n* = 44; oral swab, *n* = 56) collected from suspected EVD cases in Guinea. The specificity of this diagnostic test was 100% for both primer sets, while the sensitivity was 100% and 97.9% for the trailer and nucleoprotein primer sets, respectively, compared with a reference standard RT-PCR test. These observations suggest that our diagnostic assay is useful for identifying EVD cases, especially in the field or in settings with insufficient infrastructure.

## Author Summary

Rapid and accurate diagnostic tests are required to prevent transmission of Ebola virus disease (EVD). Nucleic acid diagnostic tests are highly sensitive and specific for detecting the virus. The RT-LAMP assay using a portable platform equipped with a battery is a useful technique for detecting the virus in the field or laboratories with limited infrastructure. In the present study, RT-LAMP assay detected *Zaire ebolavirus* in clinical specimens with diagnostic accuracies corresponding to that of the standard RT-PCR test. In addition, the assay could detect positive samples within 15 minutes. These findings have raised the prospects for developing a rapid molecular diagnostic test for EVD. With further improvements in methods for pretreatment of clinical specimens, the RT-LAMP assay may provide a highly accurate rapid diagnostic test for EVD at point-of-care in endemic rural areas.

## Introduction

The largest outbreak of Ebola virus disease (EVD) to date occurred in West Africa, mainly in Guinea, Liberia, and Sierra Leone, in 2014–2015 [1, 2]. The World Health Organization (WHO) reported 28601 cases affected by the disease and 11300 deaths up to 30 December 2015 [3]. Retrospective surveillance indicated that the outbreak was initiated by a single introduction of a strain of *Zaire ebolavirus* (EBOV) from unidentified natural reservoirs in December 2013 in the forest region of southeast Guinea. The variant of the virus introduced, EBOV Makona variant, subsequently spread by human-to-human transmission [1, 4]. Diagnosis of EVD based on clinical manifestations, especially in the early stages of infection, is very difficult as there are no specific initial symptoms of the disease [5]. Rapid laboratory detection is required for early identification of EBOV-infected patients and the provision of supportive medical care in suspected cases. In addition, a highly accurate test could reduce the risk of releasing EBOV-infected individuals into their communities and exclude potential exposure of uninfected people to EVD patients in care facilities. Therefore, a rapid and accurate laboratory diagnosis would play an important role in stemming the transmission chain of EVD [6, 7].

EBOV has a single-stranded negative-sense RNA genome that encodes seven viral proteins flanked by 3'- and 5'-terminal untranscribed sequences referred to as the leader and trailer regions, respectively [8]. Various detection techniques, such as reverse transcription PCR (RT-PCR), viral antigen-capture enzyme-linked immunosorbent assay, and immunofiltration assay, are now available as laboratory tests for the virus [9, 10]. Real-time RT-PCR (rRT-PCR) is a sensitive, specific, and reliable diagnostic technique for detecting the virus, which is used as the gold standard to identify cases of EBOV infection during outbreaks. In past and current EBOV outbreaks, rRT-PCR was employed to detect EBOV in local and mobile laboratories [5, 11]. However, rRT-PCR requires expensive equipment, a stable power supply, and skilled personnel. Total run time to obtain the results from clinical specimens, including sample inactivation and RNA extraction steps, is several hours. Regions where most outbreaks of EVD occur usually do not have sufficient infrastructure for good laboratory diagnostic services. Therefore, there is an urgent requirement for more rapid and easy-to-use diagnostic techniques for detection of the virus.

Previously, we reported a reverse transcription-loop-mediated isothermal amplification (RT-LAMP) method for the rapid detection of EBOV [12]. LAMP is a rapid and sensitive nucleic acid amplification method performed under isothermal conditions [13]. As LAMP reaction can be performed with simpler equipment compared to PCR, the RT-LAMP method for EBOV could be useful in the field or under poor laboratory settings. Current innovative sequencing technologies have revealed the genome sequences of previously isolated strains as well as current strains of EBOV circulating in West Africa [1, 14–18]. The increased information regarding EBOV genome sequences will allow improvement of the RT-LAMP assay to achieve more precise and reliable detection of the virus.

In this study, we established an RT-LAMP assay for EBOV using a novel portable LAMP detection platform. Based on the latest genetic information of EBOV strains, we improved the primers targeting the trailer region developed in our previous study, and developed new primers targeting the nucleoprotein (NP) region. Furthermore, to determine the diagnostic accuracy of this assay, we conducted an evaluation study using RNA samples obtained from sera or oral swabs collected from suspected EVD cases in Guinea.

## Methods

### Viral RNA

Viral RNA of Lassa, Marburg, and Ebola virus strains were kindly provided by Dr. Heinz Feldmann as described previously [19]. Viral RNA of EBOV H.sapiens-wt/GIN/2014/Makona-C05 (GenBank accession number, KJ660348.2) was kindly provided by Dr. Gary Kobinger, the National Microbiology Laboratory of the Public Health Agency of Canada. Viral RNAs of arboviruses, Rift Valley fever virus, dengue virus, yellow fever virus, and chikungunya virus, were kindly provided by Dr. Koichi Morita, Institute of Tropical Medicine, Nagasaki University (Nagasaki, Japan). Vesicular stomatitis virus Indiana strain and influenza A virus PR8 strain were propagated in Vero and MDCK cells, respectively, and viral RNAs were extracted from infected culture supernatants using a QIAamp Viral RNA mini kit (Qiagen, Hilden, Germany).

### LAMP primers

Nucleic acid sequence alignments of EBOV strains, including current West African strains, were constructed using ClustalX [19]. The primers for the trailer region were redesigned based on the primers developed in our previous study [12]. The primers for NP were designed with the LAMP primer design software PrimerExplorer v4 (http://primerexplorer.jp/e/) as described previously [19]. The sequences of each primer are shown in Table 1. FIP and BIP included a TTTT spacer between the F1c and F2 sequences, and the B1 and B2c sequences, respectively.

**Table 1:** Sequences of LAMP primers for EBOV.

Target	Primer	Sequence (5'-3')
trailer	F3	CAATAAACAACTATTTAAATAAC
	FIP	GTCACACATGCTGCATTGTGTTTTCTATATTTAGCCTCTCTCCCT
	BIP	AACGCAACATAATAAACTCTGCATTTTATCAATAACAATATGAGCCCAG
	B3	CTGGCAAGATATTGATACAACA
	LF	AATTTTTTGATTATCACGC
NP	F3	TGAAGTCAAGAAGCGTGATGG
	FIP	CATGGCAGCAAGTGTTCTCTTTTTAGTGAAGCGCCTTGAGGAA
	BIP	CAGTTTCTCTCCTTTGCAAGTCTTTTTGAACCTTCTCAAGGCAAGCC
	B3	AGTCCTTGCTCTGCATGTACT
	LF	TGTTTTTTCCACTAGATACTGCTGG
	LB	TCCTTCCGAAATTGGTAGTAGGA

### RT-LAMP

RT-LAMP was performed with a real-time turbidimeter (LA-200; Eiken Chemical Co. Ltd., Tokyo, Japan) or a real-time fluorescence detection platform (Genie III; OptiGene, West Sussex, UK). DEPC-treated distilled water and synthesized RNA with partial genome sequences of COD/76/Mayinga strains were used as negative and positive controls, respectively [19]. Real-time turbidity detection for LAMP amplification was carried out with a Loopamp RNA amplification kit (Eiken Chemical) at 63°C for 60 minutes in a final reaction volume of 25 μl containing 2 μl of synthesized RNA dilution. Positive results and time for positive results with this method were determined as described previously [12]. The real-time fluorescence detection of LAMP amplification was carried out with Isothermal Master Mix for Genie III (Nippon Gene, Tokyo, Japan). The reaction mixture (total volume, 25 μl) contained 15 μl of Isothermal Master Mix including GspSSD DNA polymerase and intercalator dye, 1 μl of AMV RTase (0.15 U; Life Technologies, Carlsbad, CA), 4 μl of LAMP primer mix consisting of 5 pmol of F3 and B3, 20 pmol of FIP and BIP, 10 pmol of LF and LB, and 2 μl of synthesized RNA dilution or 5 μl of RNA extract from clinical specimens. LAMP amplification and fluorescence detection were performed in Genie III at 63°C for 30 minutes, followed by dissociation analysis at 95°C–80°C for 5min. Nonspecific amplification was excluded by comparison of the melting temperature with that of the positive control (S1 Fig).

### Ethics statement

This study was conducted as part of the surveillance and public health response to stem EVD transmission in Guinea, and was approved by Nagasaki University Institutional Review Board. Whole blood samples from individuals with suspected EVD in the communities or EVD care centers, and oral swab samples from unidentified fatal cases in the communities were collected for EVD diagnostic testing and transmission surveillance as part of the activities of the National Viral Hemorrhagic Fever Project of Guinea.

### Sample collection

The samples used in this study were collected in Conakry and its surrounding districts. The collected samples were transported to the reference laboratory of Donka National Hospital (DNH) at Conakry for detection of EBOV. RNAs were extracted from sera and oral swab suspensions with a QIAamp Viral RNA mini kit (Qiagen) according to the manufacturer’s protocol. RNA samples were eluted in 60 μl of elution buffer, and stored at –80°C until use. All of the samples and individual information were managed by DNH.

### Clinical evaluation study

A total of 100 RNA samples collected between February and March 2015 were selected at random from the RNA sample library in DNH. rRT-PCR was carried out with a QuantiTect RT-PCR kit (Qiagen) and LightMix Modular Ebola Virus Zaire (2014) (TIB Molbiol, Berlin, Germany) as a reference test. Aliquots of 5 μl of RNA samples were added to 25-μl reaction mixtures. Each reaction was performed in a SmartCycler II system (Cepheid, Sunnyvale, CA) with a thermal cycle profile consisting of 42°C for 15 minutes, 95°C for 15 minutes, followed by 40 cycles of 95°C for 15 s and 55°C for 45 s. To quantify viral RNA, the standard curve described with 10-fold serially diluted synthesized RNA with partial sequences of Mayinga strains was used. The RT-LAMP test with Genie III was performed as an index test by the Nagasaki University team in DNH as described above. The Nagasaki University team was blinded to all sample information, including clinical status and reference diagnostic test results. At the end of this trial, the sample information was disclosed to the Nagasaki University team, and the results of reference rRT-PCR tests were compared to those of RT-LAMP.

### Data analysis

The precision of the estimates was determined by calculating exact 95% confidence intervals (CIs) for each test statistic. Data were entered into Microsoft Excel 2013 (Microsoft, Redmond, WA) and analyzed using MedCalc version 15.4 (SAS, Cary, NC).

## Results

We prepared two sets of LAMP primers for detection of all known EBOV strains, including West African strains isolated in the current outbreak (Table 1). Based on the viral genome information, we modified the primers targeting the 5'-untranscribed trailer region, which were developed previously and recognized 156 nucleotides with five oligonucleotide primers. We also designed primers for the NP coding sequences, which recognized 171 nucleotides with six primers. For trailer primers, only LF was introduced because no LB candidate primers improved LAMP amplification. *In silico* analysis of primer specificity revealed that the numbers of mismatches between each set of primers and viral target sequences were at most 6 or 5, and the levels of sequence identity were more than 95.97% or 97.71% for trailer and NP primers, respectively (S1 Table). To examine whether these primers can amplify virus sequences, we performed RT-LAMP using synthesized RNA with primer targeting sequences of five EBOV strains. RT-LAMP with trailer primers successfully detected not only the sequences of strain COD/76/Mayinga with two mismatches, but also strain COD/95/Kikwit with four mismatches, and GAB/96/2Nza, COD/07/9Luebo, and GIN/14/Makona-C05 with five mismatches (S2 Fig), indicating that the primers developed here can detect diverse sequences of EBOV strains. We also examined the primer specificities with other filoviruses, hemorrhagic fever viruses, influenza viruses, and arboviruses, which may potentially be found in areas where EBOV outbreaks occur and cause febrile illness. No cross-reactivity was detected in the reactions with both primer sets, suggesting that the LAMP primers are highly specific for EBOV strains (Table 2).

**Table 2:** Cross-reaction panel of RT-LAMP for EBOV.

			RT-LAMP^a^	
Family	Species	Strain	trailer	NP
*Filoviridae*	*Zaire ebolavirus*	COD/76/Mayinga	+	+
		COD/95/Kikwit	+	+
		GIN/14/Makona-C05	+	+
	*Sudan ebolavirus*		–	–
	*Reston ebolavirus*		–	–
	*Tai forest ebolavirus*		–	–
	*Bundibugyo ebolavirus*		–	–
	*Marburg marburgvirus*	Musoke	–	–
		Angola	–	–
		Ravn	–	–
*Arenaviridae*	*Lassa virus*	Josiah	–	–
		Pinneo	–	–
*Bunyaviridae*	*Rift Valley fever virus*	MP-12	–	–
*Flaviviridae*	*Dengue virus*	serotype 1	–	–
		serotype 2	–	–
		serotype 3	–	–
		serotype 4	–	–
	*Yellow fever virus*	17D	–	–
*Alphaviridae*	*Chikungunya virus*		–	–
*Orthomyxoviridae*	*Influenza A virus*	A/PR/8/34(H1N1)	–	–

^a^Viral RNA extracts were tested in an LA-200 turbidimeter using 10^6^ genome equivalent copies.

+: positive results;–: negative results.

Next, we examined the sensitivity of RT-LAMP for EBOV in two type of LAMP amplification and detection platform, i.e., LA-200 and Genie III. LA-200 detects turbidities of reaction mixtures due to insoluble byproducts of LAMP reaction, while Genie III detects increases in fluorescence intensity due to intercalator dye. To compare the sensitivity of the reaction with the two platforms, the same serial dilutions of synthesized RNA of COD/76/Mayinga strain were used for the reactions. More than 50% of the reaction with 680 copies or 64 copies of RNA yielded positive results with primers targeting trailer or NP, respectively (Table 3). The detection limits of LAMP reaction were not different between the two real-time monitoring platforms. However, detection time of RNA at each dilution point by Genie III was half of that by LA-200. Genie III could detect viral RNA within 15 or 20 minutes even with the smallest number of RNA molecules that can be detected by LAMP primers for the NP or trailer region. These results suggested that RT-LAMP performed with Genie III could be used as a rapid and sensitive diagnostic test for detecting EBOV RNA.

**Table 3:** Sensitivity and detection time of the RT-LAMP assay.

Primer	RNA(copies/test) ^a^	Turbidity detection (LA-200)		Fluorescence detection (Genie III)	
		Pos^b^	Tp (min)	Pos^b^	Tp (min)
Trailer	6800	6/6	27.0 ± 1.0	6/6	11.7 ± 1.6
	680	6/6	40.8 ± 5.9	5/6	18.3 ± 5.9
	68	0/6	–	1/6	16.2
	6.8	0/6	–	0/6	–
NP	6400	6/6	18.6 ± 0.2	6/6	11.7 ± 1.4
	640	6/6	21.7 ± 0.8	6/6	12.3 ± 1.0
	64	5/6	32.6 ± 5.6	3/6	14.0 ± 1.1
	6.4	1/6	30.9	0/6	–
	0.64	0/6	–	0/6	–

^a^Tenfold serially diluted synthesized RNA with partial genome sequences of COD/76/Mayinga strains was used in this experiment.

^b^The number of positive results per 6 reactions with diluted RNA templates.

Tp, Time for detecting positive results (mean ± standard deviation).

–, negative results.

To assess the utility of RT-LAMP assay with Genie III for clinical diagnosis of EBOV, we conducted an evaluation study using clinical samples collected from suspected EVD cases in Guinea. A total of 100 RNA samples extracted from clinical specimens were randomly selected from the sample library in DNH. The characteristics of the samples tested in this study are described in Table 4. All samples used in this study had been subjected to a reference rRT-PCR test to confirm EBOV infection. Forty-four samples were serum from suspected EVD cases. Fifty-six samples were oral swabs collected from corpses with unidentified deaths in the communities to perform post-mortem testing. The results of the RT-LAMP assay with Genie III are summarized in Table 5. Forty-seven samples were positive in the reference rRT-PCR test. The results of RT-LAMP with primers for the trailer region corresponded completely to those of the reference test. Only one rRT-PCR-positive sample was negative in RT-LAMP assay with primers for NP, and all other samples showed results corresponding to those of the rRT-PCR test. No false positives were observed in the test with both trailer and NP primers. To assess the diagnostic accuracy of the RT-LAMP assay, four statistics were determined, i.e., sensitivity, specificity, positive predictive value (PPV), and negative predictive value (NPV) (Table 5). RT-LAMP using primers for the trailer region showed values of 100% for all of these parameters. The RT-LAMP using NP primers had 100% (95% CI: 93.3–100) specificity, 97.9% (95% CI: 88.7–100) sensitivity, 100% (95% CI: 92.3–100) PPV, and 98.1% (95%CI: 90.1–100) NPV. These results suggested that the RT-LAMP assay with each primer set could be used as a highly sensitive and specific clinical diagnostic test for identifying EVD cases. The only sample that showed a negative result in this test with NP primers was an oral swab suspension collected from a corpse with a cycle threshold (C_t_) value of 36.3 in the reference test. Three samples showed higher C_t_ values, but all of these samples showed positive results in RT-LAMP with NP primers. The negative results in RT-LAMP with NP primers may have been caused by poor quality of the RNA sample, lower sensitivity of NP primers on Makona variant strains, or mutations in the primer recognition position.

**Table 4:** Clinical characteristics of the samples tested in this study.

Characteristic	Value
Cases—Sample type	
Suspected–serum	44
Corpses–oral swab	56
Total	100
Female sex no. ^a^	34/71
Age ^a^	
Range	8 months– 85 years
Mean ± SD	30.0 ± 20.4
Regions ^a^, ^b^	5

^a^ Individual information was defined for 71 of 100 cases included in this study.

^b^The samples used in this study were collected in Conakry and the surrounding prefectures, Boffa, Dubréka, Forécariah, and Mali.

**Table 5:** Diagnostic accuracy of the RT-LAMP test for EBOV compared with the reference rRT-PCR test.

	RT-LAMP^a^			
	trailer		NP	
	positive	negative	positive	negative
rRT-PCR test (n = 100)				
positive	47	0	46	1
negative	0	53	0	53
Diagnostic accuracy (95%CI)				
Sensitivity	100% (92.5–100)		97.9% (88.7–100)	
Specificity	100% (93.3–100)		100%(93.3–100)	
Positive predictive value	100% (92.5–100)		100%(92.3–100)	
Negative predictive value	100% (93.3–100)		98.1%(90.1–100)	

^a^The RT-LAMP test was performed in Genie III for a total of 100 RNA extracts from 44 sera and 56 swab suspensions.

**Fig 1:**
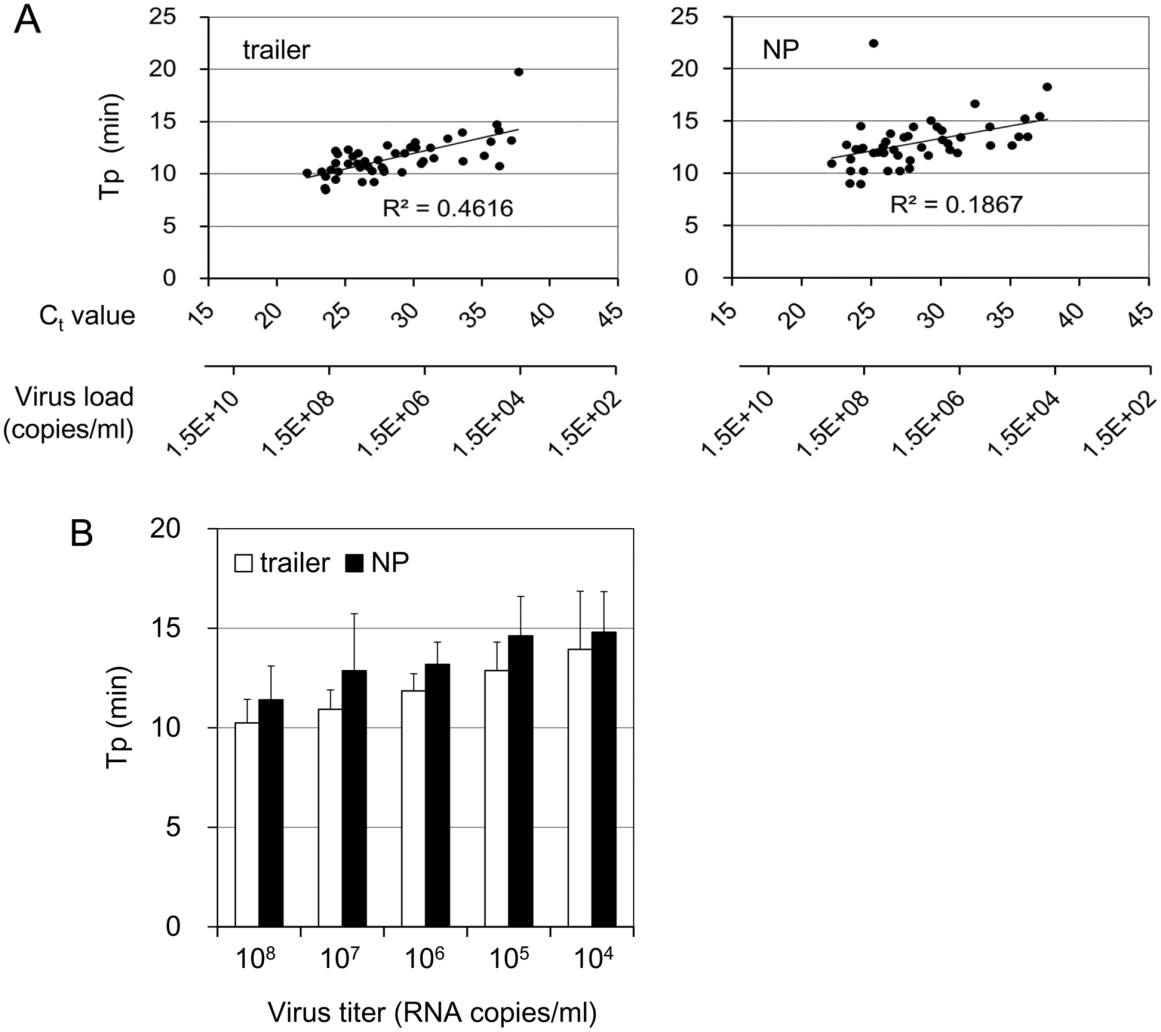
Detection time of EBOV RNA from the clinical samples with the RT-LAMP assay. (A) Tp of respective samples determined by RT-LAMP test with primers for trailer and NP were plotted against C_t_ value determined with the reference rRT-PCR test. The virus load of each sample determined by C_t_ value is indicated on the lower x-axis. (B) Mean times of detection of each group with indicated virus load were determined. White and black bars indicate the results of RT-LAMP with primers specific for trailer and NP, respectively (mean ± standard deviation).

The C_t_ values of the samples used in this study ranged from 22.3 to 37.5. The viral load of each clinical sample was determined to be in the range from 5.3×10^8^ to 1.9×10^4^ RNA copies/ml (6.2×10^6^ to 2.2×10^2^ RNA copies per reaction). The times for detection of a positive result (Tp) in this assay were 8.5–19.8 minutes with primers for trailer and 9.0–22.5 minutes with primers for NP. The C_t_ values have often been used to estimate viral load in patients. Tp from the RT-LAMP with each primer set was correlated with C_t_ values from the reference rRT-PCR test (Fig 1A; Pearson’s correlation = 0.679, *P* < 0.0001 for trailer primers, 0.432, *P* < 0.005 for NP primers). Some samples deviated from the trend line. This variation may have been due to sampling errors arising in the test of RT-LAMP or lower qualities of RNA samples used in this test. Nonetheless, the positive correlation between these tests indicated that the Tp from RT-LAMP test may be used to estimate virus load in the sample. The mean times for detecting RNA in the samples with the lowest level of viral RNA (10^4^–10^5^ copies/ml) were 13.9 minutes and 14.8 minutes with the primers for trailer and NP, respectively (Fig 1B). These results indicated that the RT-LAMP assay using a novel platform, Genie III, could determine most positive results within 15 minutes.

## Discussion

Here, we developed a rapid detection assay for EBOV by RT-LAMP using an isothermal amplification and detection platform, Genie III. This platform is a portable B5-size instrument (net weight, 1.75 kg) equipped with a battery. The RT-LAMP assay coupled with this platform would have advantages for use in field laboratories or EVD care points compared with rRT-PCR assay. Genie III also has a function to perform dissociation analysis after the DNA amplification step, which could exclude unintended amplification. The conventional LAMP detection instrument, LA-200, can monitor DNA amplification in real-time by turbidity of each reaction, but cannot exclude nonspecific DNA amplification. RT-LAMP with this platform could also represent a quicker and more accurate method of detecting EBOV. There has been an urgent requirement for rapid and high-throughput diagnostic tests in the current outbreak. rRT-PCR assay time after sample preparation is usually 45 to 90 min. On the other hand, RT-LAMP assay time was approximately 35 min including dissociation analysis. In this study, although we have monitored RT-LAMP amplification for 30 min on Genie III, most positive results could be obtained within 15 to 20 minutes even in the test with the lowest level of authentic viral RNA (Fig 1). Optimized amplification time should be determined by further study. Although some rapid tests based on rRT-PCR have also been developed, they generally take a high cost. The RT-LAMP assay can be defined by its rapidity for obtaining results, the low cost and the portability of the equipment. It could be used as a supplemental diagnostic test, especially under conditions where the gold standard, rRT-PCR, is not available.

In the current EBOV outbreak in West Africa, a rapid diagnostic test (RDT) that can be used at the bedside or in point-of-care settings has been required. Necessary characteristics of RDT include the ability for the test to be performed without pretreatment of clinical samples, no requirement for cold-chain transportation, work without electrical power, and be able to be performed by personnel without laboratory skills. Lateral flow test kits for detecting EBOV antigen have been developed for RDT [20, 21]. This molecular technology is highly valuable when considering patient triage in an overburden clinical setting. Despite lower clinical accuracy of the test, the use of RDT in early triaging of suspected patients reduced the potential risk of nosocomial transmission for health care workers and suspected patients without EBOV. Mathematical modeling estimated that the combinations of the RDT and RT-PCR test might reduce the scale of endemics of EVD by over a third [22]. Our RT-LAMP test showed clinical accuracy as high as that of the reference rRT-PCR test even in the samples with lower virus titer (maximum C_t_ 37.5). However, the RT-LAMP assay still requires pretreatment steps for sample inactivation and RNA extraction. Rapid and simple pretreatment method should also be investigated to apply the assay for EBOV point-of-care diagnostic testing.

It has been reported that the virus titer in the initial days of admission reached 10^6^–10^9^ genomes/ml in fatal cases and less than 10^5^–10^6^ genomes/ml in nonfatal cases in the cases in Sierra Leone [5]. The samples used in this evaluation study were randomly selected from the pooled RNA samples, and included 10^4^–10^8^ copies of viral RNA per milliliter (Fig 1). The samples tested in this study included possible viral titers observed in the actual clinical samples. Four of seven samples with lower virus titer (< 10^5^ copies/ml) were serum samples from suspected EVD patients. According to their clinical information, they showed only one or some of the initial symptoms of EVD, such as fever, headache, diarrhea, and conjunctival injection. These observations suggest that the RT-LAMP test could detect EBOV in samples from patients in the early stages of infection. Further evaluation studies are required to determine the detection limit of the assay with clinical samples.

We demonstrated that the RT-LAMP assay was highly specific for EBOV strains, and had no cross-reactivity with other viruses that could potentially cause hemorrhagic fever (Table 2). Although there were 5 or 6 mismatched positions in the primers for the trailer region against the viral sequences, we successfully detected the viral RNAs with diverse sequences (S2 Fig). LAMP may be rather tolerant to mismatches in the primer recognition sequences, as DNA synthesis from the primer that incompletely hybridized the target sequence could be complemented by other oligonucleotide primers. EBOV strains isolated in West Africa are phylogenetically placed in a distinct clade from Central Africa strains [1, 14]. Full genome sequence analysis identified more than 300 or 400 single nucleotide polymorphisms in the genomes isolated in Sierra Leone [14, 15]. In 2014, EVD cases were also reported in the Democratic Republic of Congo, and the isolated strains had diverse sequences even when compared with other equatorial African variants [23]. The virus sequence may mutate more frequently than estimated in natural hosts. The sequence information must be assessed to improve the primers for adaptation to strains that may emerge in future.

In summary, our assay using RT-LAMP could detect EBOV within 15 minutes using a portable instrument and showed clinical accuracy equivalent to the reference rRT-PCR test. Our test can be performed easily by personnel that are able to perform the PCR test. The Genie III portable platform is more cost-effective than rRT-PCR platforms. It would be easier to introduce the test in countries experiencing EVD outbreaks or exposed to potential risk of the disease. Our technique would also contribute to public health responses in countries with outbreaks of EVD, not only for early detection of EBOV infection but for preparedness for future outbreaks of the disease.

## Supporting Information

S1 Table Sequence identities between LAMP primers and EBOV strain sequence (DOCX)

S1 Fig A novel LAMP detection platform, Genie III. (A) Representative result of fluorescence detection by Genie III. (B) Dissociation analysis by Genie III. The dissociation curve indicated here shows the analysis results after RT-LAMP test using primers for the trailer region.
(TIF)

S2 Fig Detection of viral RNA of Central and West African strains by RT-LAMP. 10^3^ copies of artificial RNA with partial genome sequences of indicated strain (~300 nt) were amplified with RT-LAMP reaction using primers for trailer. The reaction mixtures were incubated at 63°C for 60 minutes in LA-200. After the reaction, the LAMP products were detected by agarose gel electrophoresis. 1, COD/76/Mayinga; 2, COD/95/Kikwit; 3, GAB/96/2Nza; 4, COD/07/9Luebo; 5, GIN/14/Makona-C05; 6, negative control; M, 100-bp ladder molecular maker.
(TIF)

## References

[1] Baize S, Pannetier D, Oestereich L, Rieger T, Koivogui L, Magassouba N, et al Emergence of Zaire Ebola virus disease in Guinea. N Engl J Med. 2014;371:1418–25. 10.1056/NEJMoa1404505 24738640

[2] WHO Ebola Response Team. Ebola virus disease in West Africa—the first 9 months of the epidemic and forward projections. N Engl J Med. 2014;37:1481–95. 10.1056/NEJMoa1411100 PMC4235004 25244186

[3] World Health Organization. Ebola Situation Report, 2015 December 30. Available: http://apps.who.int/ebola/current-situation/ebola-situation-report-30-december-2015

[4] Kuhn JH, Andersen KG, Baize S, Bào Y, Bavari S, Berthet N, et al Nomenclature- and Database-Compatible Names for the Two Ebola Virus Variants that Emerged in Guinea and the Democratic Republic of the Congo in 2014. Viruses. 2014:6:4760–4799. 10.3390/v6114760 25421896 PMC4246247

[5] Schieffelin JS, Shaffer JG, Goba A, Gbakie M, Gire SK, Colubri A, et al Clinical illness and outcomes in patients with Ebola in Sierra Leone. N Engl J Med. 2014;371:2092–100. 10.1056/NEJMoa1411680 25353969 PMC4318555

[6] Okeke IN, Manning RS, Pfeiffer T. Diagnostic schemes for reducing epidemic size of African viral hemorrhagic fever outbreaks. J Infect Dev Ctries. 2014;8:1148–59. 10.3855/jidc.4636 25212079

[7] Faye O, Boelle PY, Heleze E, Faye O, Loucoubar C, Magassouba N, et al Chains of transmission and control of Ebola virus disease in Conakry, Guinea, in 2014: an observational study. Lancet Infect Dis. 2015;15:320–6. 10.1016/S1473-3099(14)71075-8 25619149 PMC4373532

[8] Feldmann H, Geisbert TW. Ebola haemorrhagic fever. Lancet. 2011; 377:849–62. 10.1016/S0140-6736(10)60667-8 21084112 PMC3406178

[9] Martin P, Laupland KB, Frost EH, Valiquette L. Laboratory diagnosis of Ebola virus disease. Intensive Care Med. 2015;41:895–8. 10.1007/s00134-015-3671-y 25636586

[10] Lucht A, Formenty P, Feldmann H, Gotz M, Leroy E, Bataboukila P, et al Development of an immunofiltration-based antigen-detection assay for rapid diagnosis of Ebola virus infection. J Infect Dis. 2007;196 Suppl 2:S184–92. 17940948 10.1086/520593

[11] Onyango CO, Opoka ML, Ksiazek TG, Formenty P, Ahmed A, Tukei PM, et al Laboratory diagnosis of Ebola hemorrhagic fever during an outbreak in Yambio, Sudan, 2004. J Infect Dis. 2007;196 Suppl 2:S193–8. 17940949 10.1086/520609

[12] Kurosaki Y, Takada A, Ebihara H, Grolla A, Kamo N, Feldmann H, et al Rapid and simple detection of Ebola virus by reverse transcription-loop-mediated isothermal amplification. J Virol Methods. 2007;141:78–83. 17194485 10.1016/j.jviromet.2006.11.031

[13] Notomi T, Okayama H, Masubuchi H, Yonekawa T, Watanabe K, Amino N, et al Loop-mediated isothermal amplification of DNA. Nucleic Acids Res. 2000;28:E63 10871386 10.1093/nar/28.12.e63 PMC102748

[14] Gire SK, Goba A, Andersen KG, Sealfon RS, Park DJ, Kanneh L, et al Genomic surveillance elucidates Ebola virus origin and transmission during the 2014 outbreak. Science. 2014; 345:1369–72. 10.1126/science.1259657 25214632 PMC4431643

[15] Park DJ, Dudas G, Wohl S, Goba A, Whitmer SL, Andersen KG, et al Ebola Virus Epidemiology, Transmission, and Evolution during Seven Months in Sierra Leone. Cell. 2015;161:1516–26. 10.1016/j.cell.2015.06.007 26091036 PMC4503805

[16] Simon-Loriere E, Faye O, Faye O, Koivogui L, Magassouba N, Keita S, et al Distinct lineages of Ebola virus in Guinea during the 2014 West African epidemic. Nature. 2015;524:102–4. 10.1038/nature14612 26106863 PMC10601606

[17] Carroll MW, Matthews DA, Hiscox JA, Elmore MJ, Pollakis G, Rambaut A, et al Temporal and spatial analysis of the 2014–2015 Ebola virus outbreak in West Africa. Nature. 2015;524:97–101. 10.1038/nature14594 26083749 PMC10601607

[18] Carroll SA, Towner JS, Sealy TK, McMullan LK, Khristova ML, Burt FJ, et al Molecular evolution of viruses of the family Filoviridae based on 97 whole-genome sequences. J Virol. 2013;87:2608–16. 10.1128/JVI.03118-12 23255795 PMC3571414

[19] Kurosaki Y, Grolla A, Fukuma A, Feldmann H, Yasuda J. Development and evaluation of a simple assay for Marburg virus detection using a reverse transcription-loop-mediated isothermal amplification method. J Clin Microbiol. 2010;48:2330–6. 10.1128/JCM.01224-09 20421440 PMC2897471

[20] Walker NF, Brown CS, Youkee D, Baker P, Williams N, Kalawa A, et al Evaluation of a point-of-care blood test for identification of Ebola virus disease at Ebola holding units, Western Area, Sierra Leone, January to February 2015. Euro Surveil: 2015;20:15–20. 10.2807/1560-7917.es2015.20.12.21073 25846490

[21] Broadhurst MJ, Kelly JD, Miller A, Semper A, Bailey D, Groppelli E, et al ReEBOV Antigen Rapid Test kit for point-of-care and laboratory-based testing for Ebola virus disease: a field validation study. Lancet. 2015:386:867–874. 10.1016/S0140-6736(15)61042-X 26119838

[22] Nouvellet P, Garske T, Mills HL, Nedjati-Gilani G, Hinsley W, Blake IM, et al The role of rapid diagnostics in managing Ebola epidemics. Nature: 2015: 528:S109–16. 10.1038/nature16041 26633764 PMC4823022

[23] Maganga GD, Kapetshi J, Berthet N, Kebela Ilunga B, Kabange F, Mbala Kingebeni P, et al Ebola virus disease in the Democratic Republic of Congo. N Engl J Med. 2014;371:2083–91. 10.1056/NEJMoa1411099 25317743

