# Supplementary material for: Development and Evaluation of Reverse Transcription-Loop-Mediated Isothermal Amplification (RT-LAMP) Assay Coupled with a Portable Device for Rapid Diagnosis of Ebola Virus Disease in Guinea
Source: PLoS Negl Trop Dis. 2016 Feb 22;10(2):e0004472. doi: 10.1371/journal.pntd.0004472 (PMC4764121; doi:10.1371/journal.pntd.0004472)
Supplement: S1 Table — (DOCX) [file pntd.0004472.s001.docx]

**S2 Table. Sequence identities between LAMP primers and EBOV strain sequence.**

|  | Trailer | | NP | |
| --- | --- | --- | --- | --- |
| EBOV strain^a^ | no. mismatches | Identity (%) | no. mismatches | Identity (%) |
| COD/1976/Mayinga | 2 | 98.66 | 0 | 100.0 |
| GAB/1994/Gabon | 4 | 97.32 | 0 | 100.0 |
| COD/1995/13625 Kikwit | 4 | 97.32 | 1 | 99.42 |
| GAB/1996/1Eko | 4 | 97.32 | 0 | 100.0 |
| GAB/1996/2Nza | 5 | 96.64 | 0 | 100.0 |
| GAB/2002/Ilembe | 3 | 97.99 | 4 | 97.66 |
| COD/2007/9 Luebo | 5 | 96.64 | 0 | 100.0 |
| COD/2014/Lomela-Lokolia19 | 6 | 95.97 | 0 | 100.0 |
| GIN/2014/Gueckedou-C05 | 5 | 96.64 | 3 | 98.25 |
| GIN/2014/Gueckedou-C07 | 5 | 96.64 | 3 | 98.25 |
| GIN/2014/Kissidougou-C15 | 5 | 96.64 | 3 | 98.25 |
| SLE/2014/G3687 (SL1) ^b^ | 5 | 96.64 | 3 | 98.25 |
| SLE/2014/G3800 (SL2) ^b^ | 5 | 96.64 | 4 | 97.66 |
| SLE/2014/G3857 (SL3) ^b^ | 5 | 96.64 | 4 | 97.66 |
| LBR/2014/201403007 | 5 | 96.64 | 4 | 97.66 |
| MLI/2014/Mali-DPR1 | 5 | 96.64 | 5 | 97.08 |

^a^Accession numbers of respective strains were NC_002549, KC242792, KC242796, KC242793, KC242794, KC242800, KC242784, KP271020, KJ660348, KJ660347, KJ660346, KM034563, KM233081, KM233115, KP178538 and KP260799 in descending order.

^b^ SL1, SL2, and SL3 denote lineages of EBOV Makona variants isolated in Sierra Leone by Gire et al (*13*).
