# Supplementary figures and images for: Development and Evaluation of Reverse Transcription-Loop-Mediated Isothermal Amplification (RT-LAMP) Assay Coupled with a Portable Device for Rapid Diagnosis of Ebola Virus Disease in Guinea
Source: PLoS Negl Trop Dis. 2016 Feb 22;10(2):e0004472. doi: 10.1371/journal.pntd.0004472 (PMC4764121; doi:10.1371/journal.pntd.0004472)

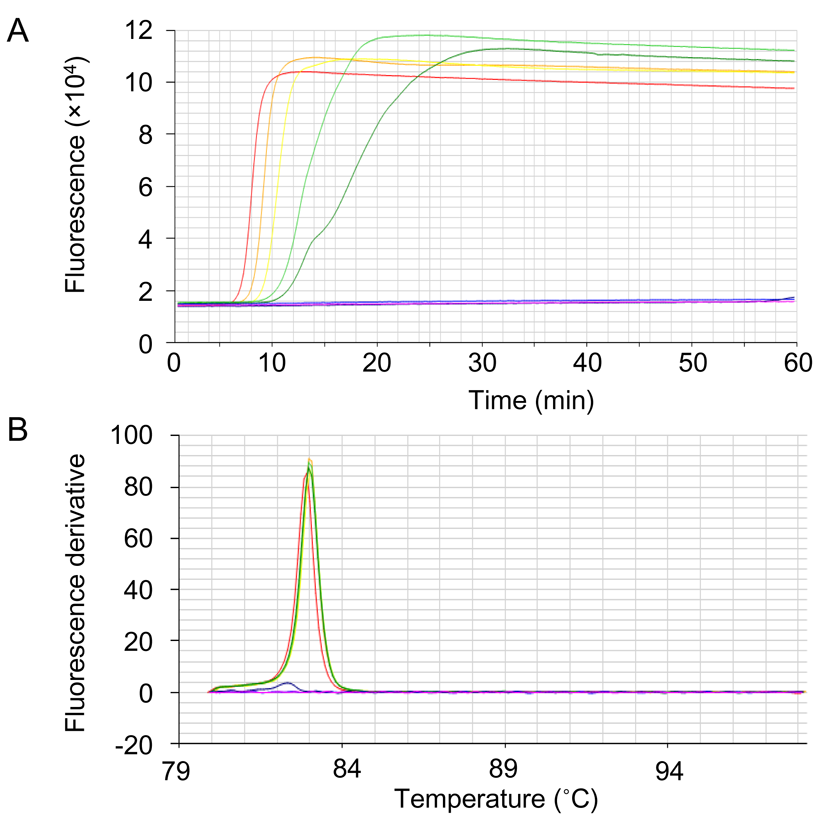

Supplement: S1 Fig — (A) Representative result of fluorescence detection by Genie III. (B) Dissociation analysis by Genie III. The dissociation curve indicated here shows the analysis results after RT-LAMP test using primers for the trailer region. (TIF) [file pntd.0004472.s002.tif]

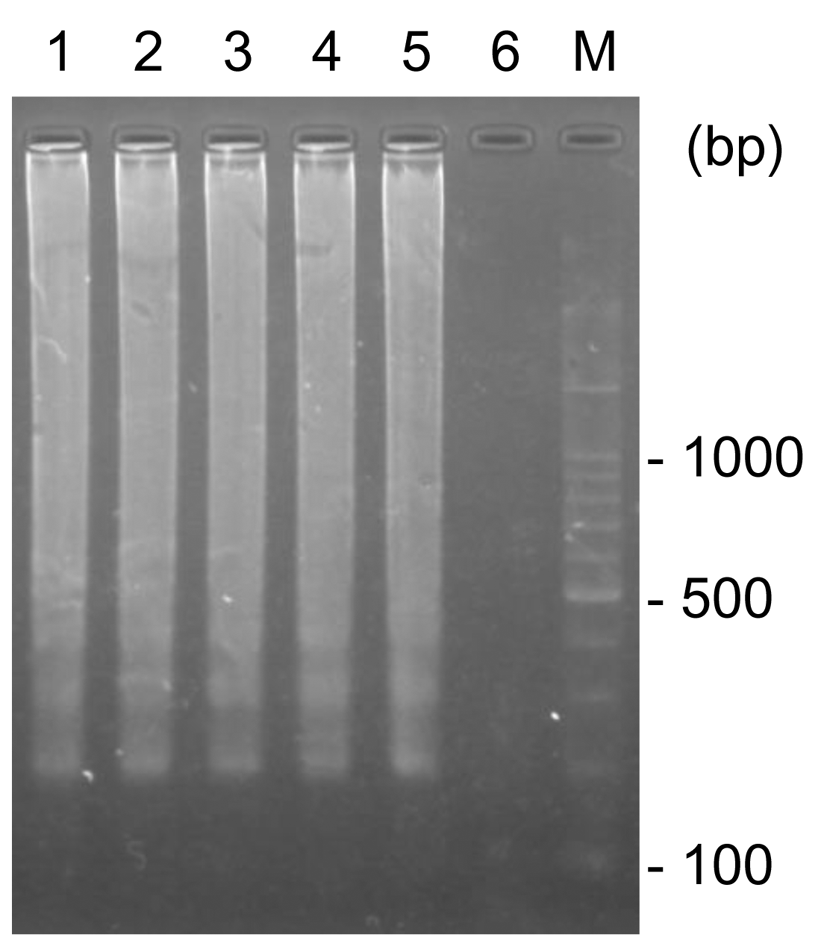

Supplement: S2 Fig — 103 copies of artificial RNA with partial genome sequences of indicated strain (~300 nt) were amplified with RT-LAMP reaction using primers for trailer. The reaction mixtures were incubated at 63°C for 60 minutes in LA-200. After the reaction, the LAMP products were detected by agarose gel electrophoresis. 1, COD/76/Mayinga; 2, COD/95/Kikwit; 3, GAB/96/2Nza; 4, COD/07/9Luebo; 5, GIN/14/Makona-C05; 6, negative control; M, 100-bp ladder molecular maker. (TIF) [file pntd.0004472.s003.tif]
